# Non-linear Embedding Methods for Identifying Similar Brain Activity in 1 Million iEEG Records Captured From 256 RNS System Patients

**DOI:** 10.3389/fdata.2022.840508

**Published:** 2022-05-20

**Authors:** Sharanya Arcot Desai, Thomas Tcheng, Martha Morrell

**Affiliations:** ^1^NeuroPace, Inc., Mountain View, CA, United States; ^2^Stanford University, Stanford, CA, United States

**Keywords:** non-linear embedding, deep learning, big data, iEEG (intracranial EEG), epilepsy, representation learning

## Abstract

Finding electrophysiological features that are similar across patients with epilepsy may facilitate identifying treatment options for one patient that worked in patients with similar brain activity patterns. Three non-linear iEEG (intracranial electroencephalogram) embedding methods of finding similar cross-patient iEEG records in a large iEEG dataset were developed and compared. About 1 million iEEG records from 256 patients with drug-resistant focal onset seizures who were treated in prospective trials of the RNS System were used for analyses. Data from 200, 25, and 31 patients were randomly selected to be in the train, validation, and test datasets. In method 1, ResNet50 convolutional neural network (CNN) model pre-trained on the ImageNet dataset was used for extracting feature maps from spectrogram images (ImageNet-ResNet) of iEEG records. In method 2, ResNet50 custom trained on an iEEG classification task using ~138,000 manually labeled iEEG records was used as the feature extractor (ESC-ResNet). Feature maps were passed through dimensionality reduction and k nearest neighbors were found in the reduced feature space. In method 3, a 256 dimensional iEEG embedding space was learned via contrastive learning by training a ResNet50 model with triplet training sets generated using within-patient iEEG clustering (CL-ResNet). All three methods had comparable performance when identifying iEEG records from the search dataset similar to test iEEG records of baseline (non-seizure) and interictal spiking activity. Epileptic interictal spikes are represented by vertical (broadband) edges in spectrogram images, and hence even generic features extracted using models trained on everyday images appear to be sufficient to represent iEEG records with similar levels of interictal spiking activity in close proximity. In the case of electrographic seizures, however, the ESC-ResNet model, identified cross-patient iEEG records with electrographic seizure morphology features that were most similar to the test iEEG records. For nuanced electrographic seizure iEEG representation learning, domain specific model training with manually generated labels had the advantage. Finally, representative iEEG records were selected from every patient using an unsupervised clustering method which effectively reduced the number of iEEG records in the search dataset from ~750,000 to 2,148, thus substantially reducing the time required for finding similar cross-patient iEEG records.

## Introduction

Identifying patients with epilepsy who share similar clinical and electrocorticographic features may help to identify effective treatment approaches (Geller et al., 2017; Jobst et al., 2017; Nune et al., 2019). For example, studies have shown that patients with mesiotemporal lobe epilepsy who have similar electrographic features have similar clinical outcomes when treated with responsive direct brain stimulation (Desai et al., 2019a,b). Another recent study in patients with epilepsy who have similar anatomical abnormalities (i.e., periventricular nodular heterotopia) found predominantly low voltage fast electrographic activity at the seizure onsets (Nune et al., 2019). In another study, grouping epileptic patients into high or low risk states based on electrographic activity helped to identify effective stimulation frequencies (Chiang et al., 2021). Collectively, these studies demonstrate the usefulness of grouping patients with similar electrographic activity, and suggest that there is an association between electrographic activity patterns, anatomical abnormalities, and effective therapy approaches.

To the best of our knowledge, a systematic study for identifying similar iEEG (intracranial electroencephalogram) patterns in a large multi-patient dataset has not been performed. Implantable neurostimulators capable of capturing chronic time-series iEEG data have been approved by the FDA only within the last decade (Skarpaas et al., 2019), and large cross-patient ambulatory iEEG datasets were previously not available. In comparison, other domains, such as e-commerce, have had access to large datasets for several years (Krizhevsky et al., 2012) which has led to the development of advanced computer vision techniques for finding similar objects (Deselaers and Ferrari, 2011; Wu et al., 2013; Wang et al., 2014). For example, e-commerce websites such as Amazon.com and Baidu.com suggest products that are visually similar to those queried by customers. Google's “Search by image” feature uses these methods to find images visually similar to an example provided by the user. In the healthcare domain, a few studies have applied analogous computer vision techniques for clustering and identifying similar images from multiple patients. For example, in one study, similar skin cancer images from multiple patients were clustered together (Esteva et al., 2017), and in another study, similar cross-patient diabetic retinopathy images were clustered together (Dondeti et al., 2020). Inspired by these studies, this study evaluates computer vision techniques for finding similar cross-patient brain activity using spectrogram images of time-series iEEG records.

One method of learning image representations for identifying similar images is through self-supervised learning (Chen et al., 2020a; Grill et al., 2020), a deep learning approach where the CNN (convolutional neural network) is trained to learn similarity metrics directly from images by leveraging underlying structure in data. For example, this may be performed using triplet training sets generated via an unsupervised clustering technique (Wang et al., 2014). Triplets contain an anchor image, a positive image and a negative image, where the positive image is more similar to the anchor image compared to the negative image. The deep learning model is trained using the hinge loss metric to minimize the Euclidean distance between the query and positive image, and to maximize the Euclidean distance between the query and the negative image (Wang et al., 2014; Chiang et al., 2021). In this way, the model's weights and biases are explicitly trained to embed similar images close to each other in an n-dimensional space. In this analysis, an unsupervised clustering method for generating iEEG triplet training examples was explored by leveraging the observation that electrographic activity patterns within a patient are generally stereotypical (Manford et al., 1996).

Another common image representation learning technique is to embed the images in a low dimensional space using pre-trained CNNs as feature extractors followed by PCA (Principal Component Analysis), and t-SNE (t-distributed stochastic neighbor embedding) or UMAP (Uniform Manifold Approximation and Projection) for dimensionality reduction (Desai et al., 2019b; Barry et al., 2021). Then, kNN (k nearest neighbors) is typically used to find neighbors nearest to the input/query images in the low dimensional space. In a previous study, we demonstrated that a similar approach works well for clustering within-patient iEEG records with long trains of abnormal epileptiform events (Barry et al., 2021). However, iEEG patterns from different patients can be very different (Haas et al., 2007), and the effectiveness of this method on cross-patient iEEG records was not yet explored. Here, we used a pre-trained ResNet50 model trained on the ImageNet dataset (ImageNet-ResNet) as a feature extractor for finding similar cross-patient iEEG records.

In contrast to using CNNs pre-trained on images from a different domain, CNNs trained on images from the same domain may serve as better feature extractors for the intended problem, since the learned features may be more relevant (Cui et al., 2018). For this reason, a recently-developed custom-trained ResNet50-based electrographic seizure classifier (ESC-ResNet) trained and tested on ~138,000 manually labeled iEEG records obtained by the RNS System (Barry et al., 2021), was explored as a feature extractor. Since this model was trained using iEEG data captured from a 113 patients, we hypothesize that it may have learned relevant cross-patients iEEG features that might transfer effectively to the task of clustering similar iEEG records across patients (Barry et al., 2021). We tested this hypothesis by using the ESC-ResNet model (Barry et al., 2021) as a feature extractor for finding similar cross-patient iEEG records with different levels of epileptic activity, and comparing its performance to a ResNet50 model trained on the ImageNet dataset.

Irrespective of the method used for finding similar patients, model inference time needs to be reasonably fast, a matter of seconds, for it to be practically useful in a production system. Since electrographic activity within a patient tends to be stereotypical (Manford et al., 1996), it may be sufficient to search through representative iEEG records in each patient, instead of searching through every iEEG record in every patient. Consequently, within-patient clustering of iEEG records was performed using pre-trained CNN features, and its performance was compared to spectral power based methods.

For several reasons, this work is unique and substantially adds to existing literature on using deep learning techniques for mining large brain activity datasets. First, it introduces the novel problem of finding similar patients based on iEEG data. Second, it discusses a generic unsupervised clustering method for identifying representative iEEG records within a patient. Third, it demonstrates the usefulness of converting time-series iEEG waveforms to spectrogram images for leveraging advances in computer vision image processing techniques. Fourth, it describes three different methods of embedding iEEG records in low dimensional spaces to identify similar cross-patient iEEG records, each method with a different level of developmental complexity. Finally, it shows that even a generically pre-trained model such as ImageNet-ResNet finds similar cross-patient iEEG records with baseline, interictal spiking, and noise events, whereas domain specific training may be needed to identify iEEG records with finer details such as patterns at the onset of electrographic seizures.

## Methods

Data for this study were obtained from the NeuroPace® RNS® System clinical trials (Nair et al., 2020). All study protocols were approved by the US FDA and the institutional review boards of the participating investigation sites. All participants gave written informed consent. The RNS System Feasibility, Pivotal and LTT studies are registered on clinicalTrials.gov (NCT00079781, NCT00264810, and NCT00572195, respectively).

### The RNS System

**Figure 1:**
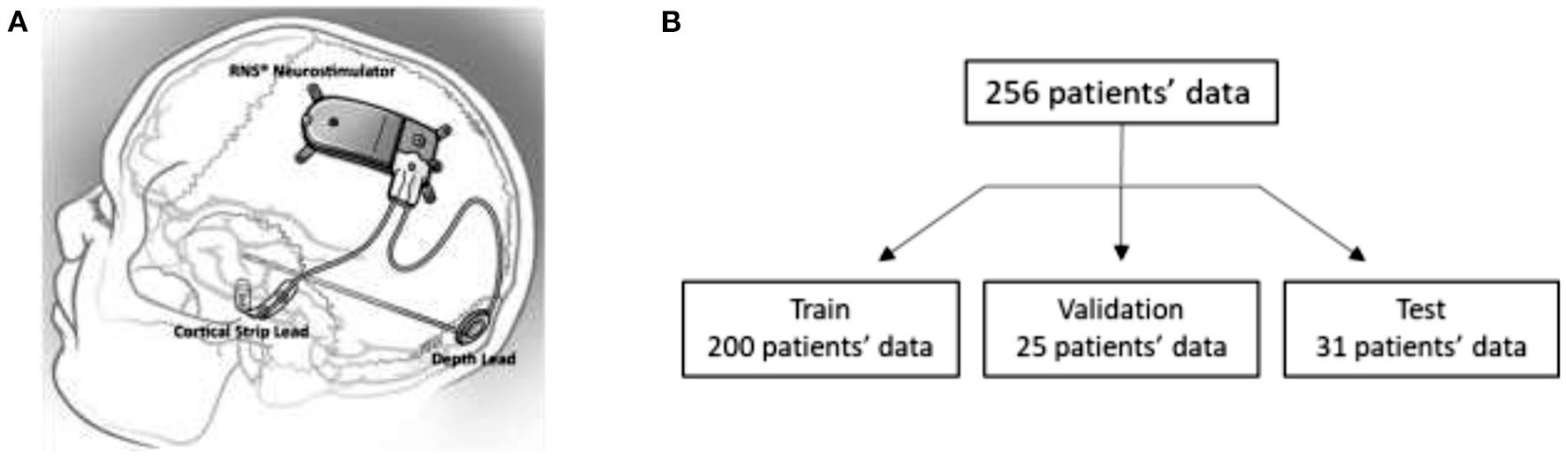
**(A)** Illustration of the NeuroPace RNS System. **(B)** Training, validation, and test data splits. iEEG records in the validation dataset were only used by one of the three methods evaluated in this paper.

Details about the RNS System are described in several previous publications (Sun and Morrell, 2014; Skarpaas et al., 2019). In brief, the RNS System is an FDA approved closed-loop responsive brain stimulation device that continuously monitors brain activity and sends electrical pulses when patient-specific abnormal patterns are detected. The device can be connected to up to 2 strip leads, or 2 depth leads, or a combination of 1 strip and 1 depth lead. Each lead contains 4 electrode contacts that are used for brain activity sensing and for delivering electrical stimulation pulses to the brain. Figure 1A shows an illustration of the NeuroPace RNS System connected to a strip lead and a depth lead.

### iEEG Records

Approximately 1 million 4-channel iEEG records have been recorded from 256 patients enrolled in the NeuroPace RNS System clinical trials (Nair et al., 2020). iEEG records typically contain 4 channels of iEEG data where each channel's data is differentially recorded between two adjacent electrode contacts on leads. The iEEG activity on each channel is recorded at 250 samples per second. The durations of iEEG records are selected by the physician and are typically 90 seconds (range: 30–240 s) long. Storage of iEEG records can be triggered by a number of different events, including detection of long trains of abnormal activity (long episode; “LE”), and pre-programmed by time-of-day (scheduled). LE iEEG records frequently contain electrographic seizures, whereas scheduled iEEG records typically contain baseline, non-seizure activity. Long episode and scheduled iEEG records together make up over 90% of all stored iEEG records. Some other types of iEEG records include “saturation” records which are stored when the sensed brain activity saturates the recording amplifiers, and “magnet” records which are stored when the patient/caregiver swipes a magnet over the implanted neurostimulator.

In the remainder of this paper, the term “iEEG record” refers to 4-channel iEEG data files captured by the RNS System, and the term “iEEG channel” refers to each channel of data in iEEG records.

### Within-Patient Data Splits

iEEG data from all 256 clinical trial patients were used for the analysis (Nair et al., 2020). A standard 60-20-20 train-validation-test split would have resulted in 164 patients in the training set. However, to increase the likelihood of finding similar cross-patient iEEG records to query iEEG records, a larger search dataset was desired; and hence, a custom data split of 78-10-12 was used. Subsequently, 200 patients' data were in the search/train dataset and 31 patients' data were in the test/query dataset. The remaining 25 patients' data were in the validation dataset and were used to guide training of the deep ranking model. In the remainder of the paper, the terms “search” and “training” are used interchangeably to refer to data from the 200 patients in the training set. The terms “query” and “test” are used to refer to data from the 31 test patients. Figure 1B shows the patient splits in the training, validation and test datasets.

### Pre-processing iEEG Records

**Figure 2:**
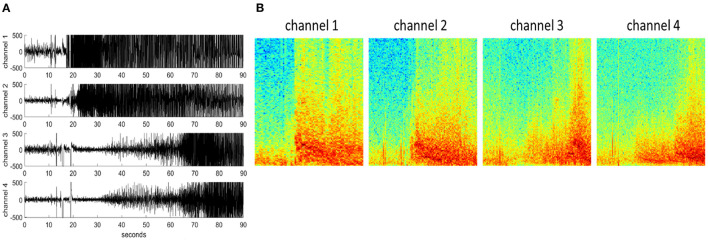
**(A)** An example 4-channel time-series iEEG record. **(B)** 224 x 224 x 3 spectrogram images of each iEEG record channel.

Each iEEG channel was converted to an RGB (3 color-channel) spectrogram image of dimensions 224 x 224 x 3 (image height x image width x number of color channels) using the methods described in a previous publication (Barry et al., 2021). Briefly, iEEG records were preprocessed to be of similar lengths (in the range of 80–100 s). iEEG records <80 s and >100 s were repeated or cropped to 90 s, respectively. iEEG records shorter than 80 s had a portion equal to the disparity duplicated from the beginning of the record and concatenated onto the beginning of the record. iEEG records longer than 90 s were cropped to include only 30 s before and 60 s after the storage trigger time. Stimulation artifact rejection was performed on all iEEG records. Matplotlib's built-in function matplotlib.pyplot.specgram with window size 256 and step size 128 was used for creating the spectrograms (0–125 Hz on the frequency axis), and saved as RBG images using the jet colormap. An example 4-channel time-series iEEG record and its corresponding spectrogram images are shown in Figure 2.

### Within-Patient Clustering to Identify Representative iEEG Records

To improve the practical usability of identifying patients with similar ECoG records, representative iEEG records were identified from each patient in the search dataset. The ResNet50 model (tf.keras.applications.resnet50.ResNet50; see TensorFlow documentation https://www.tensorflow.org/) trained on the ImageNet dataset (ImageNet-ResNet) was used to extract feature maps from the spectrogram images of each iEEG channel. The resulting feature maps (dimensions 7 x 7 x 2048) for each of the four iEEG channels were concatenated to produce a combined feature matrix (dimension 4 x 7 x 7 x 2048) for each iEEG record. Vectors of zeros were used to substitute for missing iEEG channels. Patient-specific dimensionality reduction was performed with PCA (principal component analysis) to reduce the feature matrix to the top 50 principal components for each iEEG record. Then, t-SNE (t-distributed stochastic neighbor embedding) was applied to the 50 principal components to further reduce them to two features for each iEEG record. Finally, BGMM (Bayesian Gaussian Mixture Model) clustering was applied to each patient's 2-dimensional iEEG feature sets to cluster iEEG records within each patient. The BGMM technique was selected over other clustering techniques because it automatically inferred the number of clusters within each patient. Within each cluster, the single iEEG record at the centroid of the cluster was identified. These centroid iEEG records are considered representative of the entire cluster, and iEEG records more distant from the centroid are considered less representative. The output of the within-patient clustering technique is used for creating the search dataset. Therefore, 2,148 cluster centroids from 200 patients were selected to be the search/training dataset and for generating triplets for the contrastive learning method described in Methods Section CL-ResNet Method for Identifying Similar Cross-Patient iEEG Records (Method 2).

The quality of the embedding space produced by the pre-trained ResNet50 model was explored by comparing feature maps extracted by intermediate layers of the pre-trained ResNet50 model with feature maps extracted by the final layer. Comparisons were also made with feature maps extracted by a ResNet50 model with randomly initialized parameters.

Additional embedding and clustering comparison studies were performed with spectral power features. Spectral power in seven frequency bands (0–4, 4–8, 8–12, 12–25, 25–50, 50–125, 0–125 Hz) were extracted from each iEEG channel using the scipy.signal.periodogram python function. The seven power features from each of the four iEEG channels were concatenated to produce a vector containing 28 spectral power features. Vectors of zeros were substituted for missing iEEG channels. PCA and t-SNE were applied to the resulting spectral power features within each patient. Finally, BGMM was used to cluster the 2-dimensional feature sets in each patient. Centroid iEEG records were identified for these clusters, as well.

### ImageNet-ResNet for Identifying Similar Cross-Patient iEEG Records (Method 1)

**Figure 3:**
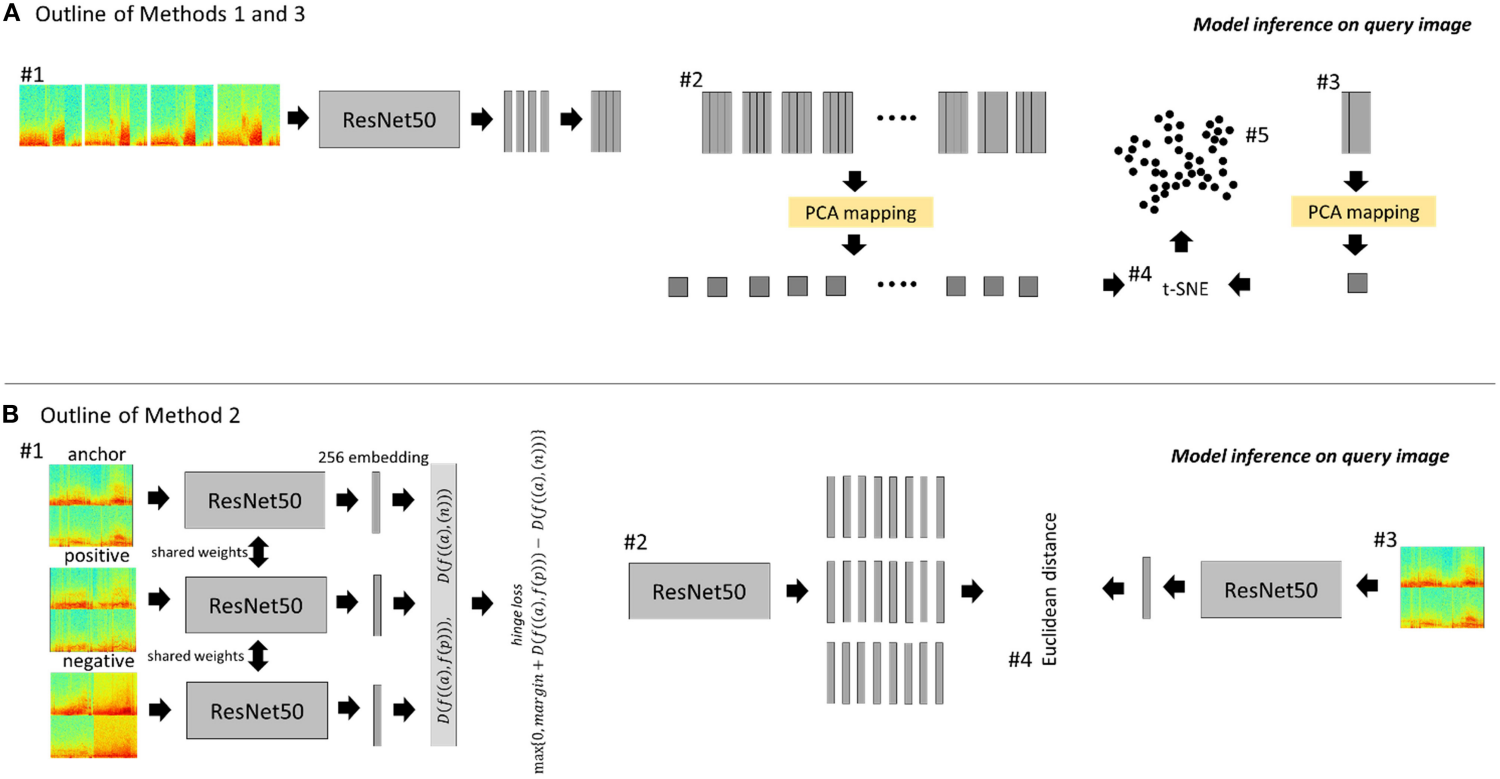
Outline of methods for identifying similar cross-patient iEEG records. **(A)** ESC-ResNet and ImageNet-ResNet methods. #1: Each channel of a time-series iEEG record is converted to a 3-color channel spectrogram image. The spectrogram images are passed through a CNN feature extractor. #2: The resulting features from all search iEEG records are analyzed by PCA. The resulting PCA mapping function is saved. #3: At inference time, the feature map of the query image and its channel permutations are passed through the previously saved PCA mapping function. #4: The 50 principal components of all search images and the query image (and its channel permutations) are passed through t-SNE. #5: K-nearest neighbors is used to find the 5 iEEG records closest to the query image and its channel permutations. **(B)** CL-ResNet method. #1: Spectrogram images of 4-channel iEEG records are tiled to form a single 224 x 224 x 3 spectrogram image for each iEEG record. Triplets were used to train a deep ranking model using the hinge loss function to embed similar images closer than dissimilar images. #2: The embeddings of all search images are generated. #3: At inference time, the embeddings of the query iEEG record and its channel permutations are generated. #4: The five highest ranked iEEG records from the search dataset are identified by computing the Euclidean distance between the search embeddings and query embeddings.

Methods Section Within-Patient Clustering to Identify Representative iEEG Records describes using the ImageNet-ResNet model for embedding within-patient iEEG records, however the performance of this model on embedding iEEG records captured from multiple patients is yet to be explored. To this end, the ImageNet-ResNet50 model was used for identifying similar cross-patient iEEG records (Figure 3A). Note that feature extraction and dimensionality reduction was performed on all iEEG records from each of the 256 RNS System patients (i.e., PCA was performed once in each of the 256 patients); in this section, on the other hand, the feature extraction and dimensionality reduction steps were performed on representative cross-patient iEEG records in the search dataset (i.e., PCA was performed once on 2,148 ECoGs from 200 patients). Additionally, unlike in Section Within-Patient Clustering to Identify Representative iEEG Records, where BGMM clustering was performed on the reduced feature sets within each patient for identifying representative within-patient iEEG records, in this section k-nearest neighbors was used instead for identifying similar cross-patient iEEG records. To elaborate, first, the pre-trained ImageNet-ResNet50 model was used to extract features from spectrogram images of iEEG channels from cluster centroids (*n* = 2,148) of the 200 patients in the training/search dataset. The resulting features were processed by principal component analysis (PCA), returning 50 principal components. The resulting PCA model was saved for use during model inference.

During inference, the spectrogram images from a test/query iEEG record and its channel permutations were passed through the saved PCA model. The resulting 50 principal components from each training/search centroid iEEG record, the query iEEG record, and its channel permutations were reduced to two dimensions using t-SNE.

K-nearest-neighbors method (python function: *sklearn.neighbors*) was used to find the 5 neighbors nearest to the test/query iEEG record and its channel permutations. The final ranking order for each test/query iEEG record was determined by ranking the search results for the original query/test iEEG record and all its channel permutations, and selecting the top 5 unique training/search iEEG records.

### CL-ResNet for Identifying Similar Cross-Patient iEEG Records (Method 2)

In method 2, a ResNet50 model was trained via contrastive learning using a triplet loss function as shown schematically in Figure 3B. Images used for this method were tiled (2 x 2) spectrograms (112 x 112 x 3) of the four channels in each iEEG record, resulting in a single image of dimension 224 x 224 x 3 representing all four channels. Triplets of training data consisting of an anchor image, a positive image and a negative image were generated for each cluster centroid iEEG record from the 200 training patients. The goal of training was to learn an image embedding function such that similar images are embedded closer than dissimilar images (Cui et al., 2018). This can be mathematically expressed as:


$$\begin{array}{l} D\left(f\left(a\right),\;f\left(p\right)\right)<D\left(f\left(a\right),\;\left(n\right)\right), \end{array}$$

Here, *D* is the Euclidean distance between the two points, *f* is the embedding function to map the image to a vector, *a* is the anchor image, *p* is the positive image, *n* is the negative image.

The hinge loss for the triplet is defined as:


$$\begin{array}{l} l\left(a,\;p,\;n\right)=\\ {}[10pt]\max\left\{0,\;margin+D\left(f(\left(a\right),\;f\left(p\right))\right)-\;D\left(f(\left(a\right),\;\left(n\right))\right)\right\} \end{array}$$

Where *l* is the hinge loss for the triplet, *margin* is a gap parameter that regularizes the gap between the distance of the two image pairs (*a, p*) and (*a, n*). The model is optimized to achieve *D*(*f*((*a*), *f*(*p*)) ~ 0 and *D*(*f*((*a*), (*n*)) > *D*(*f*((*a*), *f*(*p*))+*margin*.

Triplets can be classified into three types depending on the embedding of the negative image with respect to the positive and anchor images.

(1) An easy triplet has a hinge loss values of zero because the embedding for the negative image is further away from the anchor than the positive image by a distance greater than the *margin*, i.e., *D*((*f*(*a*), *f*(*p*)))+*margin* < *D*(*f*((*a*), (*n* )) ).
(2) A hard triplet has a positive hinge loss value because its negative image is closer to the anchor than the positive image, i.e. *D*(*f*((*a*), (*n*))) < *D*(*f*((*a*), *f*(*p* )) ).
(3) A semi-hard triplet has a positive loss value because the negative image is further from the anchor than the positive image, but not by more than the *margin* value, i.e., *D*(*f*((*a*), *f*(*p*))) < *D*(*f*((*a*), *f*(*n*))) < *D*(*f*((*a*), *f*(*n*)))+ *margin*

**Figure 4:**
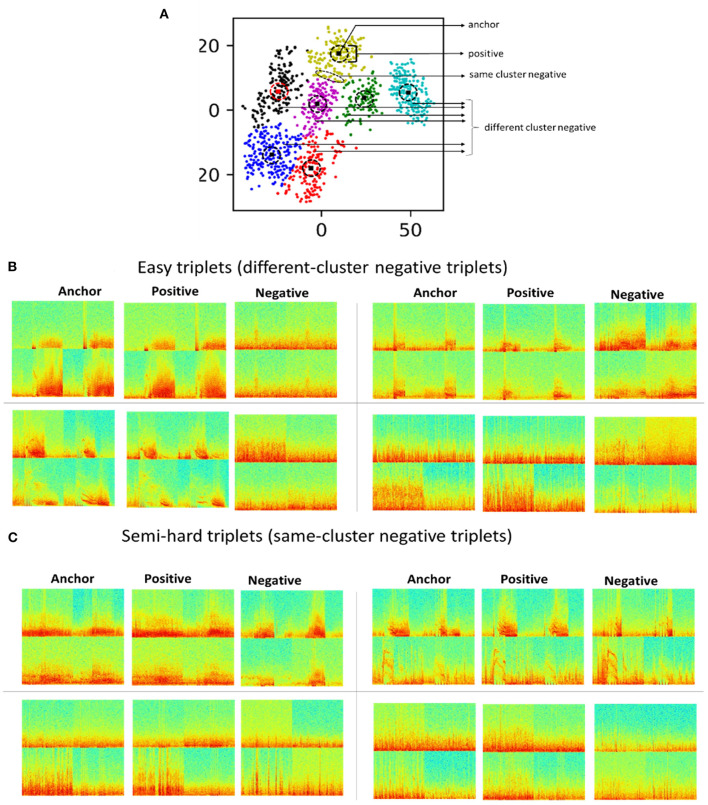
**(A)** 2D embeddings of all iEEG records from one example patient. All cluster centroid iEEG records were selected as anchors. iEEG records closest to the anchor were selected as positive images. iEEG records further away from the centroid in the same cluster were selected as negative images. iEEG records from other clusters within the same patient were also selected as negative images. **(B)** Example triplets with negative images from different clusters. **(C)** Example triplets with negative images from the same cluster.

Triplet selection had a major impact on model training, with experiments demonstrating successful training with semi-hard triplets. Inspired by previous studies (Parkhi et al., 2015; Schroff et al., 2015; Yu et al., 2018), and to include a combination of easy and semi-hard triplets, same-cluster (semi-hard) and different-cluster (easy) triplets were generated from within-patient 2D embeddings of iEEG records generated using method 5. All cluster centroid iEEG records were selected as anchors. For each anchor, same-cluster triplets were generated by selecting the three iEEG records closest to the anchor as positive images, and for every anchor and positive image pair, five more distant iEEG records in the same cluster were selected as negative images. Different-cluster triplets were generated using the same anchors, the five iEEG records closest to the anchor as positive images, and for every anchor and positive image pair, ten iEEG records in other clusters within the same patient were selected as negative images. Figure 4A shows within-patient clusters of iEEG records in one example patient. Arrows indicate regions within the 2D embedding space from which iEEG records for positive, easy negative and semi-hard negative were selected for one cluster centroid anchor iEEG record. Figures 4B,C shows example different-cluster and same-cluster triplets generated for this patient.

A number of training hyperparameters were explored including length of the final image embedding vector (range 2–256), batch size of training (range 8–32) and learning rate (range 10^−3^-10^−6^). Different and same cluster training examples generated from the 25 patients in the validation dataset were used to determine the number of training epochs, and for guiding the selection of training hyperparameters.

### ESC-ResNet for Identifying Similar Cross-Patient iEEG Records (Method 3)

In method 3, a ResNet50 model previously trained to classify iEEG channel spectrogram images into seizure and non-seizure classes was used. Detailed methods for developing the electrographic seizure classifier (ESC) are described in Barry et al. (2021). Briefly, iEEG channels in 138,000 iEEG records from 113 patients were manually labeled as seizure and non-seizure. Six different CNN model architectures were trained using five folds of training, validation, and test datasets with 72, 18 and 23 patients, respectively. A ResNet50 based model produced the highest iEEG channel classification accuracy of 95.7% (F_1_ score: 94.3%). This ESC-ResNet model was used as feature extractor in method 3 of the current study for finding similar cross-patient iEEG records.

Cluster centroids in 200 patients in the training dataset were selected for precomputing a PCA model. The ESC-ResNet model was used to extract features from spectrogram images of iEEG channels in the cluster centroids (*n* = 2,148). The feature vectors from all four channels in each iEEG record were concatenated to form an iEEG record-level feature matrix which was passed through a PCA computation step. The resulting PCA model was saved for use during model inference on the test/query data. Model inference was performed using the steps shown in Figure 3A.

Table 1 summarizes the development effort for the 3 methods.

**Table 1:** Development effort for the 3 non-linear iEEG embedding methods explored.

	**Method 1 (ImageNet-ResNet)**	**Method 2 (CL-ResNet)**	**Method 3 (ESC-ResNet)**
Labeling effort	None	None	~4 months
Amount of training data	None	Number of centroids = 2,148 Number of triplets used for training = 31,750	Number of seizure class examples = 108,277 Number of non-seizure class examples = 108,277
Code development effort	Minimal: Existing model used as feature extractor.	Moderate: Generating positive and negative training examples for triplet datasets. Model training, model architecture exploration, and hyperparameter optimization.	Moderate: Developing training and validation datasets. Model training, model architecture exploration, and hyperparameter optimization.

### Query Image Selection and Channel Permutations

The performance of three methods for finding similar cross-patient iEEG records for 10 query/test iEEG records with varying levels and types of epileptic activity is shown in Table 2. Each method returned a ranked list of similar iEEG records from patients in the training/search dataset when presented with an iEEG record from a patient in the query/test dataset. Table 2 summarizes the activity patterns observed in each of 10 query iEEG records. Query iEEG records 1–6 contain electrographic seizure activity or clear epileptiform activity on one or more iEEG channels. Query record 7 contains interictal spiking activity. Query records 8 and 9 contain baseline non-seizure electrographic activity. Query record 10 contains frequent stimulation artifact on all four iEEG channels. The top five ranked iEEG records returned by each method from the training/search dataset for the 10 query iEEG records are shown in the Section Results.

**Table 2:** Brief descriptions of the 10 test/query iEEG records.

**Query iEEG record number**	**Short description of activity**
1	Electrographic seizure with long (~35 s duration) high frequency band (50–75 Hz) on all 4 channels. Ictal activity more prominent on channels 1 and 4, compared to 2 and 3.
2	Electrographic seizure with short (~15 s duration) high-frequency band (50–75 Hz) on channels 1, 2 and 3. Increased spectral power at relatively lower frequency (<50 Hz) on channel 4.
3	Electrographic seizure with prominent frequency harmonics in spectral power near the seizure onset on all 4 channels.
4	Interictal spiking activity on channels 1 and 2, electrographic seizure activity on channels 3 and 4 with harmonics at frequencies <50 Hz.
5	Short burst of high amplitude activity on all 4 channels
6	Electrographic seizure on all 4 channels with harmonic bands throughout the seizure, especially on channels 1 and 2. Seizure termination on all 4 channels.
7	Interictal spiking activity on all 4 channels
8	Baseline activity on all 4 channels
9	Baseline activity on channels 1 and 2, very low amplitude broadband baseline activity on channels 3 and 4.
10	Evident stimulation artifact on all 4 channels.

**Figure 5:**
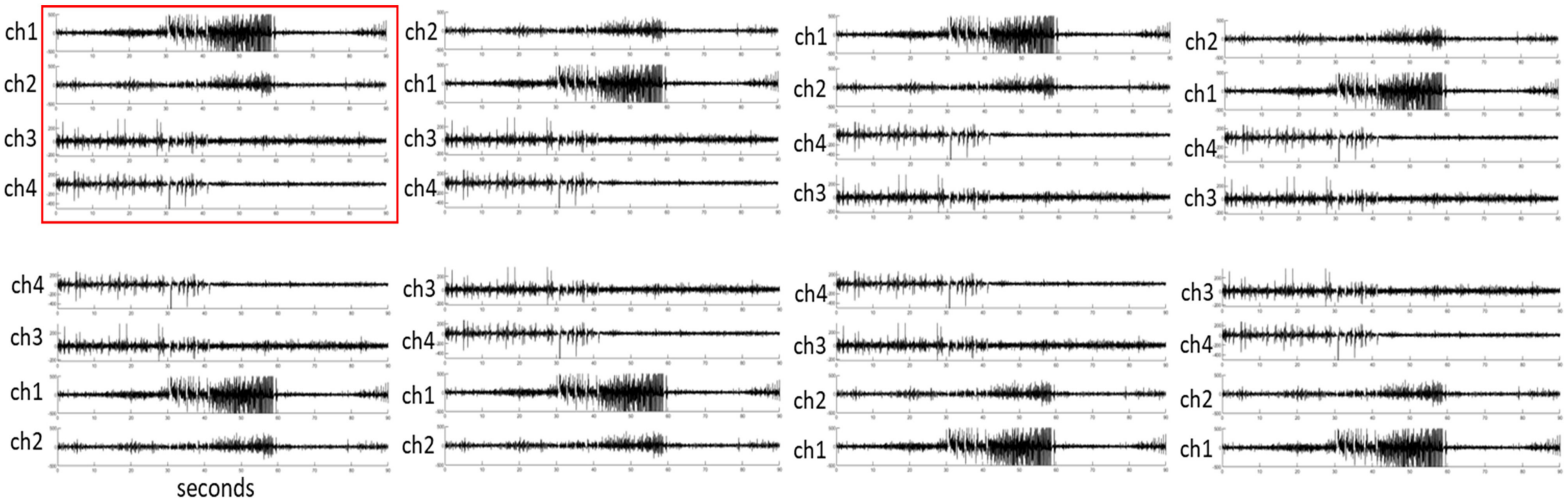
An example iEEG record (highlighted in red) and its seven iEEG channel permutations.

Because the training and test images were vertically concatenated to produce four-channel iEEG spectrogram images, and similar iEEG records could have similar channels in any order, channel permutations were performed for each test iEEG record, and images based on all 8 permutations (including the original iEEG record) were used. These equivalent images were used to search the centroid iEEG records from the 200 search patients. An example iEEG record and its seven other channel permutations are shown in Figure 5.

### Training and Inference Hardware and Software

An n1-standard-4 virtual machine with Nvidia Tesla K 80 on Google Cloud Platform was used for running all training and inference experiments described in this paper. Python 3 with Tensorflow 2.0 was used for training and inference code development.

## Results

### Finding Within-Patient Representative iEEG Records

**Figure 6:**
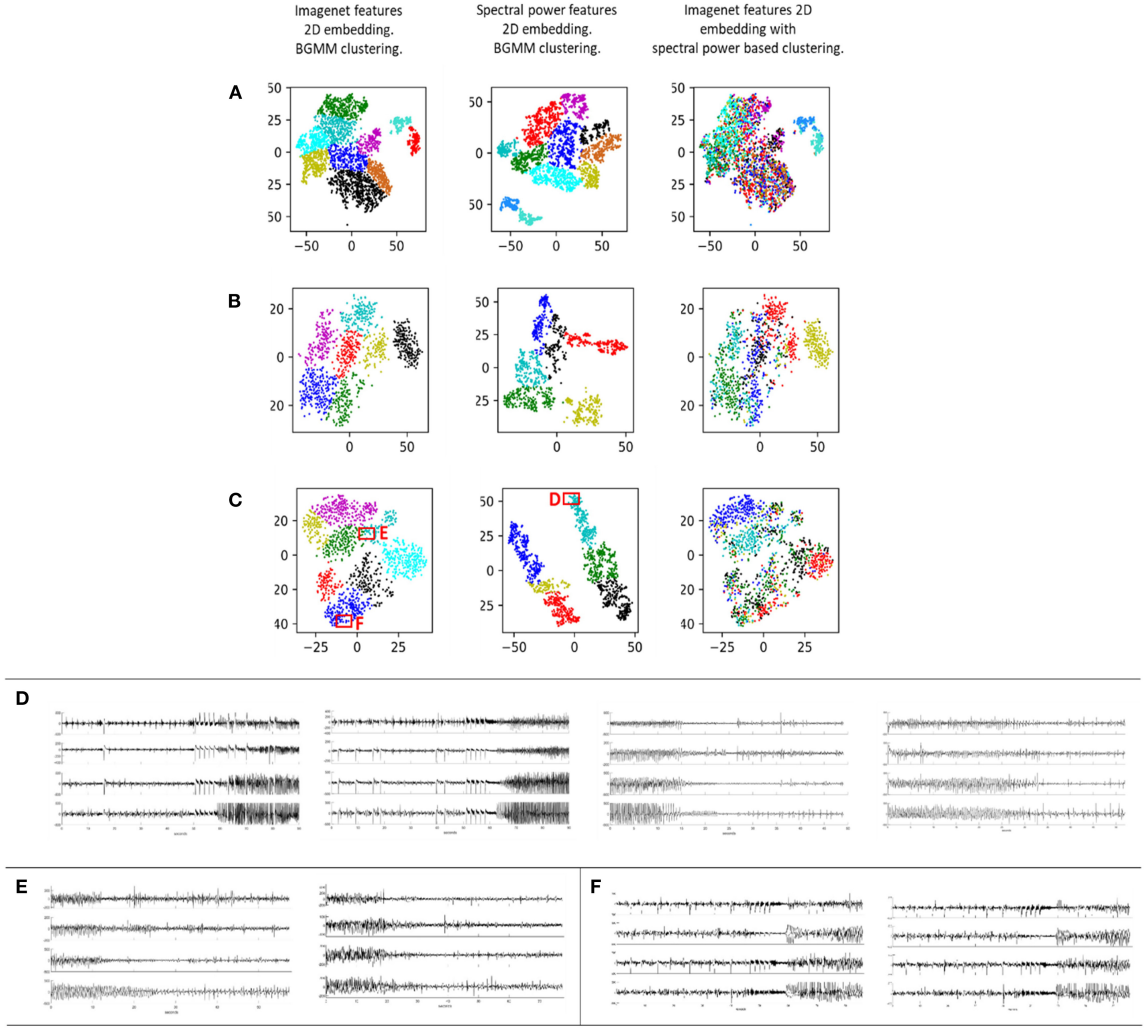
Each row **(A–C)** contains iEEG records from one individual patient represented in a 2 dimensional space. Column 1: ImageNet-ResNet was used as the feature extractor. The resulting features were passed through a dimensionality reduction technique consisting of PCA followed by t-SNE. Bayesian Gaussian Mixture Models (BGMM) was then applied to find clusters in the 2-dimensional dataset. Column 2: Spectral power in seven frequency bands was extracted and these features were passed through dimensionality reduction and clustering. Column 3: Overlap between the approaches in columns 1 and 2 is demonstrated by showing the 2-dimensional representation from column 1 with cluster membership colors from column 2. **(D)** Examples of iEEG records clustered together in box D using the spectral power feature extraction method. These records are spectrally similar but visually different. **(E,F)** Example iEEG records from two clusters in boxes E and F generated using the ImageNet-ResNet feature extraction method.

iEEG features extracted using a CNN pre-trained on a generic image dataset (ImageNet) was found to produce meaningful clustering of within-patient interictal and ictal iEEG records (Figures 6A–C column 1). The ImageNet-ResNet model, which presumably extracts points, edges and other generic non-linear patterns, was sufficient to distinguish the finite number of distinct electrographic seizure morphologies captured within individual patients. Further, clustering with a pre-trained ResNet50 model (ImageNet-ResNet) far outperformed spectral power-band based clustering. Comparison of iEEG clustering with the ImageNet-ResNet feature maps and spectral power features in three example patients are shown in Figures 6A–C. The first column shows the output of the BGMM clustering method using ImageNet-ResNet, the second column shows the output of BGMM clustering in the same patient, but with spectral power features instead of ImageNet-ResNet features. The third column shows the overlap between the two clustering methods, with ImageNet-ResNet based 2 dimensional embedding and spectral power based cluster color coding.

With spectral power based clustering, iEEG records with similar spectral power content but completely different iEEG patterns were often embedded close to each other in the 2-dimensional space. For example, Figure 6D shows iEEG records in one patient in whom electrographic seizure onsets and offsets were embedded close to each other (Figure 6, box D). However, in the same patient, ImageNet-ResNet features resulted in better embedding of iEEG records, with visually different seizure onsets embedded in different clusters (Figure 6E and box E; Figure 6F and box F).

**Figure 7:**
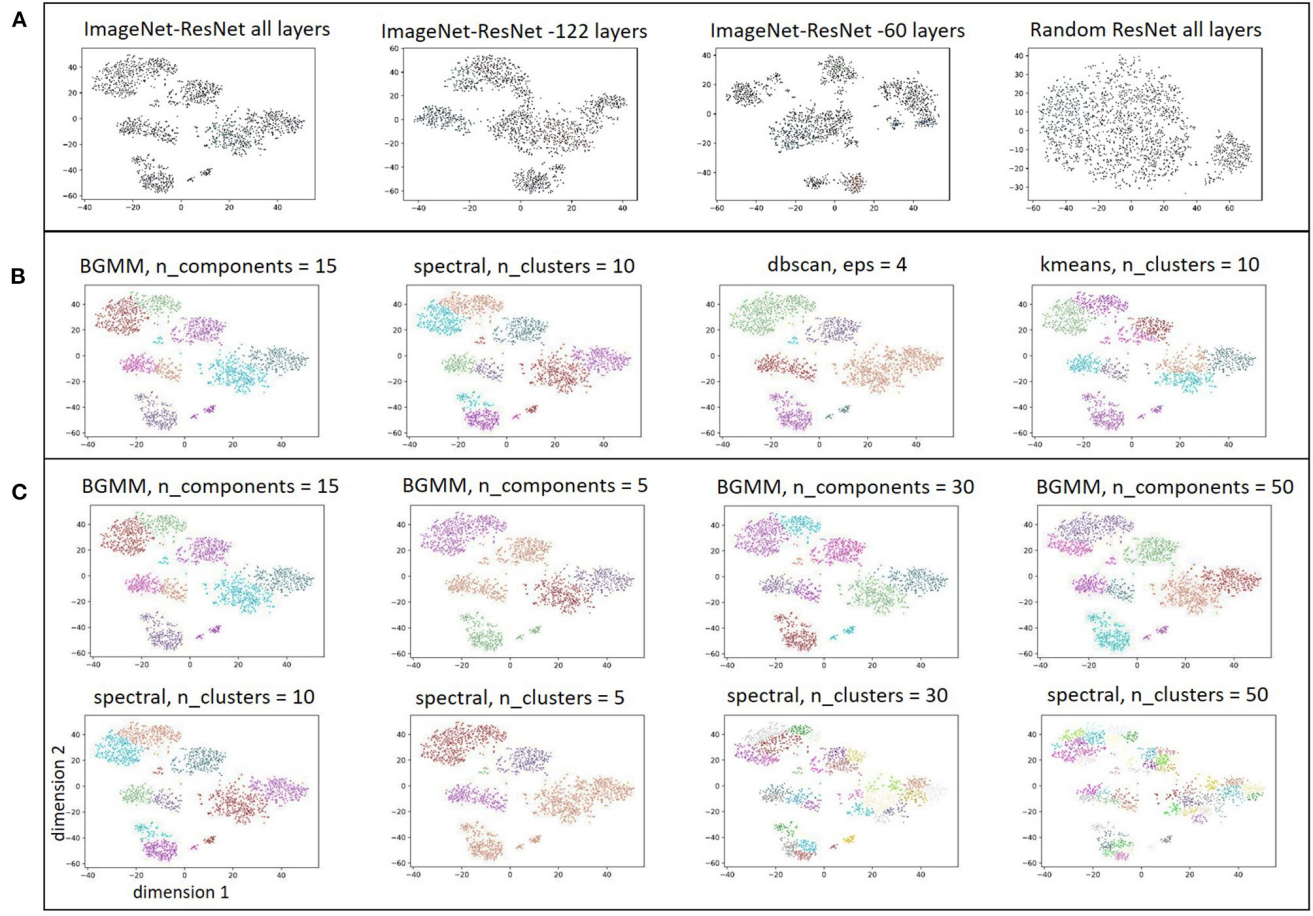
**(A)** Embedding of iEEG records in one patient with feature maps extracted by ImageNet-ResNet model containing all layers, ImageNet-ResNet with 122 layers removed from the end, ImageNet-ResNet with 60 layers removed from the end, and a ResNet50 model with randomly initialized weights. **(B)** Comparison of clusters identified by BGMM method with input parameter, n_components, set to 15, spectral clustering method with n_clusters set to 10, dbscan with epsilon set to 4, and kmeans with n_clusters set to 10. **(C)** Comparison of clusters identified by BGMM method with a range of input parameter values (n_components = 15,5,30,50) and spectral clustering method with n_clusters = 10,5,30,50. Note that spectral clustering here does not refer to clustering with hand-engineered spectral power features as discussed in Figure 6, rather it refers to a clustering method that uses a normalized Laplacian (see sklearn.cluster.spectralclustering). Comparable clustering with the dbscan method (epsilon = 4,2,3,5), and kmeans clustering method (n_clusters = 10,5,30,50) are shown in the Supplementary Figure S2.

Since it appeared that generic CNN features were able to distinguish within-patient iEEG activity, iEEG record embedding produced using a randomly initialized ResNet50 model was studied to examine if features extracted by an untrained CNN can also produce meaningful clusters. A ResNet50 model initialized with random weights and biases returned feature maps that produced clusters with substantially worse separability compared to the pre-trained ResNet50 model (Figure 7A) indicating that CNN training is necessary for producing reasonable spectrogram image embeddings.

Clusters produced from feature maps extracted using the ImageNet-ResNet at intermediate depths (i.e., with 122 layers, and 60 layers respectively removed from the end of the model) are also shown in Figure 7A. In most patients, the intermediate layers did not appear to produce better cluster separability compared to feature maps extracted using all layers of ImageNet-ResNet. Hence, all layers of ImageNet-ResNet model were used for extracting feature maps from the train (*n* = 200), test (*n* = 35) and validation (*n* = 21) patients. Clusters produced using the BGMM method were robust to the value of input parameter (n_components), provided that the value of n_components was set to be sufficiently high. While the other clustering techniques (spectral clustering, dbscan, and kmeans) produced clusters comparable to BGMM when initialized with appropriate parameters (Figure 7B), the clusters returned by the other methods were very sensitive to the input parameters (Figure 7C and Supplementary Figure S2). Since it is not feasible to manually determine appropriate input parameters in each of the 256 patients, the BGMM technique was used in all patients with the input parameter, n_components, set to be equal to the maximum of (number of iEEG records in the patient divided by 500, and 15). For example, in a patient with 2,000 iEEG records, n_components would be set to be 15; and for a patient with 20,000 iEEG records, n_components would be set to be 40. The BGMM method would determine the appropriate number of clusters in the patient, a number ≤ n_components.

Using the ImageNet-ResNet feature maps, 2,759 clusters of iEEG records were identified within the 256 patients. Cluster centroids in 200 (*n* = 2,148 cluster centroids), 31 (*n* = 337) and 25 (*n* = 274) randomly selected patients formed the training, test and validation datasets respectively. The numbers and shapes of clusters differed vastly from patient to patient, with the minimum, maximum and median number of clusters across the 256 patients being 1, 30, and 12 respectively.

### CL-ResNet Model Training and Validation Loss

Contrastive learning was sensitive to training hyperparameters, with small changes to hyperparameter values having substantial effects on the model training behavior. In particular, changes to the batch-size parameter had a large influence on model training. After exploring a range of hyperparameter values, the best results were a batch size of 16 triplets (or 48 iEEG records), embedding vector size of 256, learning rate of 10^−5^. Every layer of the ResNet50 model with ImageNet features was allowed to train. Weights and biases of all layers of the ResNet50 model changed after training. The training and validation loss dropped rapidly over the first few training epochs, demonstrating model learning that generalized to held-out patient's data. Training was stopped at 43 epochs because no improvements in validation loss were observed after this point. Supplementary Figure S1 shows changes in training and validation loss over training epochs.

### Top 5 Ranked Search iEEG Records for 10 Query iEEG Records

**Figure 8:**
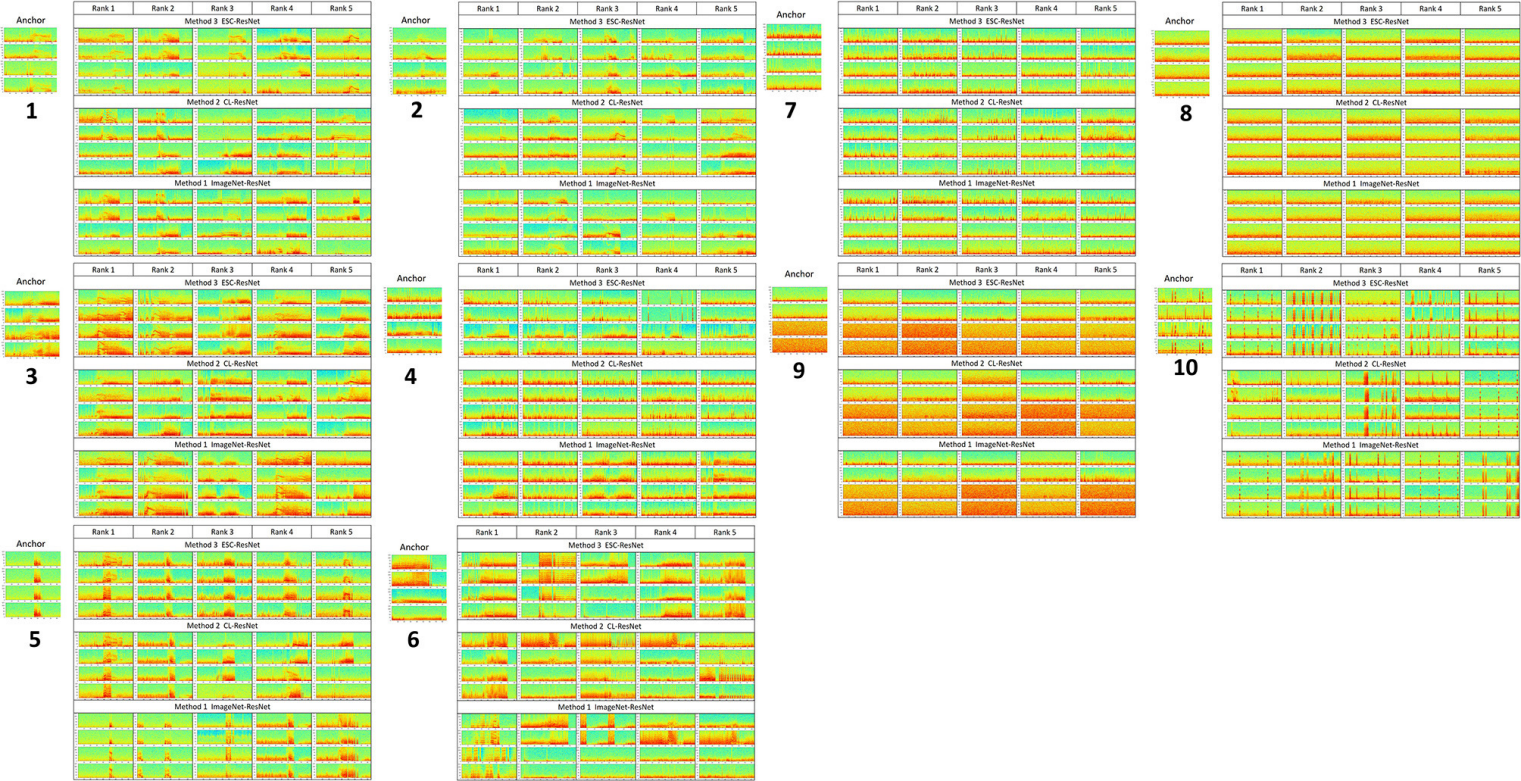
The 5 highest ranked iEEG search records for each of the 10 query iEEG records and their channel permutations.

Figure 8 shows the top five iEEG records returned by each of the three methods evaluated in this paper. For test/query iEEG records that contained electrographic seizures and short bursts of epileptiform activity (test/query records 1–6), the ESC-ResNet method generally returned results with nuanced seizure morphology features that looked most similar to the query image.

In the query iEEG records with baseline and interictal spiking activity, on the other hand, all three methods returned equivalent results.

A qualitative description of the query results returned by each of the 3 methods is provided below.

Query 1: Bands of high frequency activity between 50 and 75 Hz were present in all 5 results returned by ESC-ResNet, with the shape of the high frequency bands being similar to band in the query. With the CL-ResNet, rank 3 did not have a clear high frequency band. With the ImageNet-ResNet model, ranks 4 and 5 did not have clear high frequency bands.

Query 2: Short bands of high frequency activity were seen in all top-ranked results returned by ResNet-ESC and CL-ResNet. With ImageNet-ResNet, search ranks 1 and 5 did not contain such activity.

Query 3: Electrographic seizure activity with strong harmonics at seizure starts, similar to query, were observed in all search results returned by ESC-ResNet. Harmonics at seizure starts were not observed in 1 (rank 2) and 2 (rank 3 and 5) iEEG records returned by CL-ResNet and ImageNet-ResNet models respectively.

Query 4: ESC-ResNet returned substantially better results for the query with spiking activity on channels 1 and 2, and electrographic seizure activity with evident bands on channels 3 and 4. All 5 iEEG records returned by the ESC-ResNet were similar to the query. The CL-ResNet model returned iEEG records with interictal spiking on all four channels, whereas the ImageNet-ResNet model returned one iEEG record with electrographic seizure activity on channels 3 and 4. The other four results were dissimilar to the query.

Query 5: Short bursts of epileptiform activity was returned by all three methods. However, the activity was most similar in iEEG records returned by ESC-ResNet. Rank 5 returned by CL-ResNet did not have the bursting activity on channels 3 and 4. Rank 5 returned by ImageNet-ResNet contained electrographic seizure activity on all four channels, and the bursting activity seen in rank 1 and 2 looked different from the query.

Query 6: Harmonics in electrographic seizure with abrupt ending, similar to query, was seen in ranks 4 and 5 returned by ESC-ResNet, with ranks 1–3 containing abrupt seizure beginnings. The CL-ResNet model returned electrographic seizure with harmonics similar to the query, but 4/5 results did not have abrupt seizure endings. Out of the three methods, search results returned by the ImageNet-ResNet model looked least similar to the query.

Query 7: iEEG records with interictal spiking activity were returned by all three methods.

Query 8: iEEG records with baseline activity were returned by all three methods.

Query 9: iEEG records with baseline activity on channels 1 and 2, and very low amplitude baseline activity on channels 3 and 4 were returned by all three methods.

Query 10: iEEG records with evident stimulation artifact were returned by all three methods.

## Discussion

The work in this paper is unique and significant for several reasons. (1) Identifying patients with similar brain activity patterns may have important clinical applications such as identifying potential treatment options that are effective in similar patients. In a previous study (Wu and Gotman, 1998), hand-crafted features and string matching techniques were explored for identifying similar electrographic seizures in a small EEG dataset (with 7 electrographic seizures) from five patients (three with intracerebral electrodes, and two with scalp electrodes). Although several high-level concepts are shared between the previous study and the present study (including within-patient clustering of EEG), the previous study was limited by the amount of data used for developing the methods. To the best of our knowledge, a systematic study for identifying similar cross-patient iEEG records in a large multi-patient iEEG dataset (from >100 patients) has not been previously performed. (2) Brain activity patterns within patients are often stereotypical (Manford et al., 1996). Hence, searching a relatively small number of representative iEEG records per patient will speed up the process of identifying similar cross-patient iEEG patterns in large multi-patient iEEG datasets, making the resulting search tool more useful in production environments. Manually selecting representative iEEG records would be subject to selection bias, with different humans picking different sets of representative iEEG records. This research presents a fully unsupervised method of selecting within-patient representative iEEG records using pre-trained CNNs. This approach results in a more comprehensive and objective use of brain activity features in iEEG spectrograms to select representative iEEG records. The viability of this method was demonstrated by comparing the clustering performance of pre-trained CNN features with hand-engineered spectral power features. (3) Three different methods of identifying cross-patient iEEG records were discussed. The first method, ImageNet-ResNet, used a pre-trained CNN trained on the generic ImageNet dataset as the feature extractor. The second method, CL-ResNet, involved training a ResNet50 model with triplets generated using an unsupervised within-patient iEEG clustering technique. The final method, ESC-ResNet, involved using a custom pre-trained ResNet50 model as feature extractor. The search rankings of the three methods were compared on 10 query images.

The ESC-ResNet method, in which a custom-trained ResNet50 model was used as a feature extractor, empirically outperformed the other two methods on query images containing electrographic seizures. The ResNet50 used in this method was trained on 108,000 seizure spectrogram images and 108,000 non-seizure spectrogram images from 72 patients, with the trained model having a classification accuracy of 95.7% and F_1_ score of 94.3% on iEEG spectrogram images from held-out test patients (Barry et al., 2021). Additionally, gradient-based saliency maps revealed that the model learned relevant iEEG features, with pixels associated with electrographic seizures clearly highlighted when spectrogram images were correctly classified as seizures. The features learned by this model transferred effectively to the task of embedding similar cross-patient iEEG records with electrographic seizures close to each other in a low-dimensional space (Cui et al., 2018). This is demonstrated by the visually similar search results returned by the ESC-ResNet method even on complex query images containing two channels of interictal spiking activity and two channels of electrographic seizure activity. The superiority of this method with query images containing electrographic seizures may be attributed to the fact that a large number of electrographic seizure iEEG records from 72 patients were used for training. Even though it has been theorized and shown in computer vision tasks that models trained on auxiliary tasks serve as good feature extractors for solving other problems in the same or a similar domain (Cui et al., 2018), this is first study to systematically demonstrate it on human brain recordings by converting iEEG records to spectrogram images.

These findings should encourage future studies to convert time series physiological recordings to spectrogram images to leverage image-based CNN techniques for solving similar cross-patient data clustering problems.

In image recognition tasks where the objective is to find similar everyday objects such as cars, lamps, and human faces, training using a deep ranking approach with triplets consisting of anchor, positive and negative images, has produced better results than using pre-trained CNNs trained on generic datasets as feature extractors (Wang et al., 2014; Schroff et al., 2015; Chen et al., 2020a,b). These findings motivated the contrastive learning method explored in the present study with triplets of iEEG spectrogram images generated from time-series iEEG records. Even though reductions in training and validation losses were observed during the training process (Supplementary Figures S1,S2), this method failed to produce better results than using an electrographic seizure classifier as feature extractor in electrographic seizure query images. Marginal to moderate improvement in search results were seen compared to the baseline approach of using a ResNet50 pre-trained on the generic ImageNet dataset as feature extractor.

A few modifications to the training process could possibly improve the CL-ResNet model's performance. First, in many previous studies, CL-ResNet model training was performed using triplet images generated from well-defined, human-labeled classes, with anchor and positive images selected from within the same class, and negative images selected from a different class (Wang et al., 2014). To emulate this, spectrogram images of iEEG records within patients were clustered using pre-trained CNNs as feature extractors. For easy triplets, all cluster centroids were selected as anchors, with close neighbors within the same cluster selected as positive images and images from different clusters within the same patient selected as negative images. For semi-hard triplets, the same anchors and positive images were used, but same-cluster images further away from the anchor than the positive images were selected as negative images. Semi-hard triplets, where positive images embed closer to the anchor than the negative with a positive loss function, have been shown to produce the best training results (Parkhi et al., 2015; Schroff et al., 2015; Yu et al., 2018). Future studies should explore training using only semi-hard triplets. One way of achieving this could be through an online triplet generation technique where triplets are not pre-selected before training, but are generated during the training process (Yu et al., 2018). Second, the CL-ImageNet training process was very sensitive to hyperparameter selection, with small changes in certain hyperparameters (for example, the batch size) leading to substantial differences in model training and validation performance. Even though a range of hyperparameters was explored, the unexplored hyperparameter space is still very large. Future studies could use automated hyperparameter exploration techniques such as AutoML to more efficiently search the hyperparameter space (He et al., 2021). Third, since in epilepsy, the query images will likely contain electrographic seizures, the number of triplets with electrographic seizure iEEG records used for training could be increased. An electrographic seizure classifier could be used to guide the generation of triplets (Barry et al., 2021). Finally, in this study only within-patient triplets were used for training because it was straightforward to cluster patient-specific iEEG records. In future studies cross-patient triplets could also be included. The ESC-ResNet method, for example, could be used for generating cross-patient triplets. In this case, for every query image, the first few ranked iEEG records from other patients could be used as positive images, and iEEG records with lower ranks could be used as negative images.

Even though the ESC-ResNet method outperformed the other two methods on electrographic seizure query images, it is noteworthy that the iEEG records returned by all three methods were comparable in similarity to the query iEEG records containing baseline, interictal spiking, and stimulation artifact. Since interictal spiking activity and stimulation artifact are essentially transformed to broadband vertical edges in spectrogram images, the problem of finding cross-patient iEEG records with similar levels of interictal spiking activity and stimulation artifact is essentially a computer vision problem of identifying similar numbers and widths of distinct vertical edges in 2 dimensional images. We hypothesize that the ImageNet-ResNet model, trained on over 10 million images in 1,000 categories of everyday objects and animals, has learned to successfully identify edges and other generic patterns. Hence, the ImageNet-ResNet model is able to identify iEEG records with similar levels of interictal epileptiform events. However, domain specific training with manually labeled iEEG records appears to be important for finding cross-patient iEEG records with similar finer spectrogram-image details such as the presence of high frequency oscillation bands at the onset of epileptic seizures. In summary, if the goal is to search through large multi-patient datasets to find iEEG records with visually similar noise or interictal spiking activity, a CNN trained on a generic image dataset such as ImageNet could suffice.

Hybrid methods combining the results of the three methods could be explored in future studies. In cases where one of the three methods fails to find similar iEEG records, the top 5–10 ranked iEEG records by different methods could be displayed to the end-user who could then select visually similar iEEG records produced by all three methods. A hybrid approach could also be useful for weeding out outliers that are highly ranked by only one of the methods.

Ultimately, this effort demonstrates that it is possible to identify epilepsy patients whose iEEGs are similar to other patients and that this can be achieved in a computationally efficient way. Currently, patients with epilepsy are treated empirically. The typical course for a patient with severe focal onset seizures is one antiepileptic medication after another, followed by a lengthy evaluation to determine whether brain resection or laser ablation could be of benefit and, if neuromodulation is selected, years of exploring a variety of stimulation parameters. In the RNS System trials of patients with drug-resistant focal epilepsy, the average patient had endured 20 years of ineffective treatment (Nair et al., 2020). The potential clinical application coming from this work is that complex electrophysiological multi-patient clinical and iEEG data sets could be utilized to identify patients whose epilepsies are similar, evaluate the response in each to previous therapies, and thus, identify therapeutic approaches most likely to be effective, sparing the patient of decades of therapeutic trials and disappointments.

## Data Availability Statement

The data presented in the study are deposited in Data Archive Brain Initiative (DABI) at https://dabi.loni.usc.edu/dsi/000012.

## Ethics Statement

All study protocols were approved by the US FDA and the Institutional Review Boards of the participating investigation sites. The patients/participants provided their written informed consent to participate in this study. The RNS System Feasibility, Pivotal and LTT studies are registered on clinicaltrials.gov (NCT00079781, NCT00264810, and NCT00572195, respectively).

## Author Contributions

SA designed the study, performed the experiments, and primarily wrote the manuscript. TT and MM provided guidance during study and manuscript development. All authors contributed to the article and approved the submitted version.

## Conflict of Interest

SA, TT, and MM are employees of NeuroPace.

## Publisher's Note

All claims expressed in this article are solely those of the authors and do not necessarily represent those of their affiliated organizations, or those of the publisher, the editors and the reviewers. Any product that may be evaluated in this article, or claim that may be made by its manufacturer, is not guaranteed or endorsed by the publisher.
