# Supplementary material for: Non-linear Embedding Methods for Identifying Similar Brain Activity in 1 Million iEEG Records Captured From 256 RNS System Patients
Source: Front Big Data. 2022 May 20;5:840508. doi: 10.3389/fdata.2022.840508 (PMC9163709; doi:10.3389/fdata.2022.840508)
Supplement: Supplementary file 1 [file Data_Sheet_1.pdf]

Supplementary Material

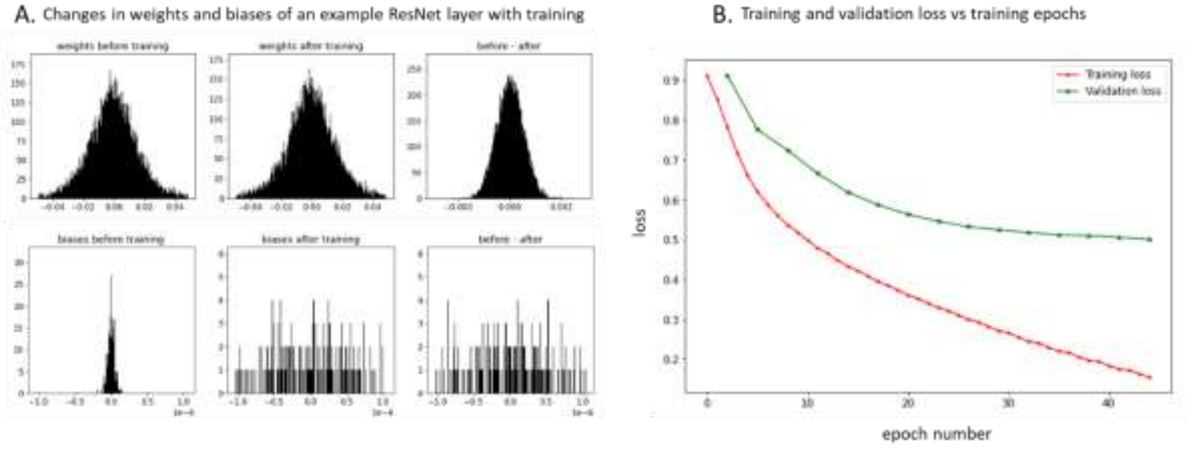

Figure S1: A. Weights and biases change after training. The first column shows a histogram of weights and biases of ResNet50 (CL-ResNet) model's 23rd layer at the beginning of training, the second column shows these values at the end of training (epoch 43), and the third column shows the difference in weights and biases at the beginning and end of model training. B. The training and validation loss both decrease as training proceeded. While the training loss reduces sharply after epoch 20, only modest improvements in validation loss is seen after this point. Training was stopped at epoch 43 because the validation loss did not improve after this point.

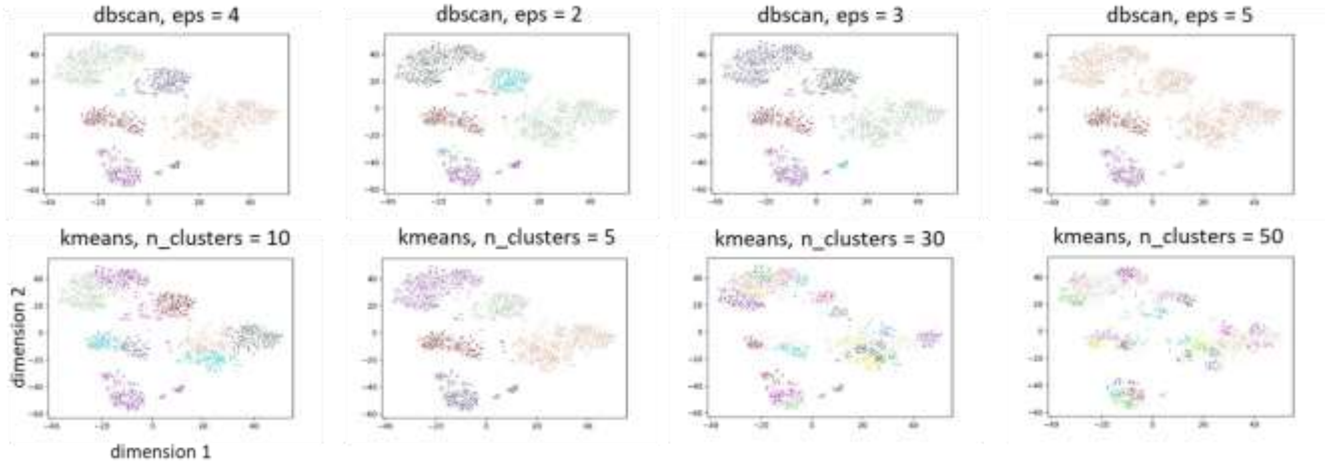

Figure S2: Clustering of the same patient's data shown in Figure 7 B and C with kmeans and dbscan clustering methods.
